# Interruption of Endolysosomal Trafficking After Focal Brain Ischemia

**DOI:** 10.3389/fnmol.2021.719100

**Published:** 2021-09-28

**Authors:** Kurt Hu, Bhakta Prasad Gaire, Lalita Subedi, Awadhesh Arya, Hironori Teramoto, Chunli Liu, Bingren Hu

**Affiliations:** ^1^Department of Medicine, Division of Pulmonary and Critical Care, Medical College of Wisconsin, Wisconsin, WI, United States; ^2^Department of Anesthesiology and Neurology, Shock Trauma and Anesthesiology Research Center, University of Maryland School of Medicine, Baltimore, MD, United States; ^3^Veterans Affairs Maryland Health Center System, Baltimore, MD, United States

**Keywords:** brain ischemia-reperfusion injury, membrane trafficking, N-ethylmaleimide sensitive fusion protein, cathepsin B, late endosome, early endosome, autophagosome, autophagic flux

## Abstract

A typical neuron consists of a soma, a single axon with numerous nerve terminals, and multiple dendritic trunks with numerous branches. Each of the 100 billion neurons in the brain has on average 7,000 synaptic connections to other neurons. The neuronal endolysosomal compartments for the degradation of axonal and dendritic waste are located in the soma region. That means that all autophagosomal and endosomal cargos from 7,000 synaptic connections must be transported to the soma region for degradation. For that reason, neuronal endolysosomal degradation is an extraordinarily demanding and dynamic event, and thus is highly susceptible to many pathological conditions. Dysfunction in the endolysosomal trafficking pathways occurs in virtually all neurodegenerative diseases. Most lysosomal storage disorders (LSDs) with defects in the endolysosomal system preferentially affect the central nervous system (CNS). Recently, significant progress has been made in understanding the role that the endolysosomal trafficking pathways play after brain ischemia. Brain ischemia damages the membrane fusion machinery co-operated by N-ethylmaleimide sensitive factor (NSF), soluble NSF attachment protein (SNAP), and soluble NSF attachment protein receptors (SNAREs), thus interrupting the membrane-to-membrane fusion between the late endosome and terminal lysosome. This interruption obstructs all incoming traffic. Consequently, both the size and number of endolysosomal structures, autophagosomes, early endosomes, and intra-neuronal protein aggregates are increased extensively in post-ischemic neurons. This cascade of events eventually damages the endolysosomal structures to release hydrolases leading to ischemic brain injury. Gene knockout and selective inhibition of key endolysosomal cathepsins protects the brain from ischemic injury. This review aims to provide an update of the current knowledge, future research directions, and the clinical implications regarding the critical role of the neuronal endolysosomal trafficking pathways in ischemic brain injury.

## Introduction

The endolysosomal system is a major catabolic pathway for recycling cellular waste products. Newer evidence has placed this system at the center stage of many research fields. This system has previously been viewed as a relatively static acidic compartment containing hydrolytic enzymes for cellular waste degradation, but now has emerged as a highly dynamic organelle capable of sensing and controlling multiple cellular processes (Repnik et al., 2013, 2015; Inpanathan and Botelho, 2019). The endolysosomal system has sophisticated organizations constantly sensing diverse intra- and extracellular stimuli to adjust their numbers, size, and position, as described in detail in several recent reviews (Condon and Sabatini, 2019; Lawrence and Zoncu, 2019; Ballabio and Bonifacino, 2020). In addition to degradation of waste protein aggregates, damaged organelles, and extracellular materials, this system plays a key role in inflammatory and immune responses, antigen presentation, plasma membrane repair, exosome release, cellular adhesion and migration, extracellular matrix (ECM) remodeling, programmed cell death, tumor invasion and metastasis, and neurodegeneration (Wang et al., 2018; Winckler et al., 2018; Inpanathan and Botelho, 2019; Lie and Nixon, 2019; Alu et al., 2020; de Araujo et al., 2020; Giovedi et al., 2020; Navarro-Romero et al., 2020; Qureshi et al., 2020; Song et al., 2020).

A typical neuron consists of a soma, a single axon with numerous nerve terminals, and multiple dendritic trunks with numerous branches. On average, there are 7,000 distal synaptic connections from each of the hundreds of billions of neurons present in the human brain to other neurons. The neuronal endolysosomal degradation compartments are located in the soma region. That means that all autophagosomal and endosomal cargos from 7,000 synaptic connections must be transported to the soma region for degradation. Therefore, the degradation of cargo in polarized neurons by endolysosomal compartments is a rigorous event, vulnerable to many pathological conditions.

In this review, we focus on the neuronal endolysosomal pathway after stroke. We provide a basic overview of updated knowledge on membrane trafficking from the endocytic and autophagic pathways to the endolysosomal compartments. Next, we illustrate the features of neuronal endolysosomal trafficking. Finally, we explore the current knowledge of dysfunctional endolysosomal compartments in animal models of brain ischemia.

## Part 1: Endocytic and Autophagic Trafficking to the Endolysosomal Compartments

### Endocytic Pathway

The primary function of the endocytic pathway is recycling and bulk degradation of internalized materials and redundant cellular components (Repnik et al., 2013). The endocytic pathway encompasses three membrane compartments: the early endosome, recycling endosome, and late endosome. This pathway begins with invagination of a segment of the cell surface membrane, typically coated with proteins such as clathrin or caveolin, to form endocytic vesicles. The endocytic vesicles either fuse with existing early endosomes to deliver their cargo or undergo multiple rounds of homotypic fusion to generate a new early endosome (Rizzoli et al., 2006). The early endosome serves as the primary sorting compartment to distribute the engulfed cargos to the trans-Golgi network, the recycling endosome, and the late endosome. Engulfed cargos include lipids, membrane proteins, signaling molecules, and extracellular substances.

In the recycling route of the endocytic pathway, the early endosome converts a part of its structure into a recycling endosome to reassemble engulfed membrane proteins for reuse back in plasma membranes (Hyttinen et al., 2013). In the degradation route, the early endosome undergoes a maturation process to become a late endosome or fuses with an existing late endosome to deliver the cargo (Hyttinen et al., 2013). The transition between early and late endosome requires switching proteins, e.g., exchanging Rab5 with Rab7, is accompanied by structural changes, such as forming of the intraluminal membranous vesicles (ILV) (Poteryaev et al., 2010). The late endosome itself also undergoes a series of transformations and comes in various forms, such as the multivesicular body (MVB) (Bissig and Gruenberg, 2013). During the transformation process, the late endosome receives newly synthesized hydrolytic enzymes and structural components from the trans-Golgi network and degradative cargos from the endocytic and autophagic (see below) pathways (Repnik et al., 2013; Bright et al., 2016). Therefore, the late endosome is often akin to the stomach of the cell. At the final mature stage, the late endosome fuses with a terminal lysosome to become an endosome–lysosome hybrid, i.e., endolysosome, where the endocytosed materials are broken up into basic components, such as amino acids, for cell reuse (Bright et al., 2016). After degradation of cargo, the endolysosome converts to a terminal lysosome in a process referred to as lysosome re-formation (Bright et al., 2016).

### Autophagy Pathway

Macroautophagy, herein referred to as autophagy, is a cellular bulk degradation mechanism to recycle intracellular waste protein aggregates and damaged organelles and is essential to maintain cell homeostasis (Liu et al., 2010). Autophagy begins with the nucleation of a membrane sack termed phagophore. This nucleation process requires multiple autophagy-related (ATG) protein and lipid complexes, such as the ATG7-ATG12-ATG16 complex, as well as the microtubule-associated protein 1A/1B-light chain 3 (LC3I)-phosphatidylethanolamine (PE) conjugate referred to as LC3II. The phagophore engulfment of waste ubiquitinated protein aggregates and damaged organelles is mediated by adaptor proteins known as autophagic receptors, such as p62/sequestosome-1 (SQSTM1), optineurin, neighbor of BRCA1 gene 1 (NBR1), and ubiquilin-2. These autophagic receptors share a common ubiquitin-binding domain (UBD) and an LC3-interacting region (LIR). They use the UBD to recognize ubiquitinated protein aggregates or ubiquinated proteins on damaged organelle membranes, and the LIR to interact with LC3II on the phagophore membrane (Deng et al., 2017). The most widely studied autophagy receptor is p62/SQSTM1. The phagophore expands during engulfment and eventually seals both ends to give rise to a double-membrane autophagosome. The autophagosome then fuses with the late endosome to form an intermediate organelle, termed amphisome. Next, the amphisome undergoes membrane fusion with a terminal lysosome to become an autolysosome where cargo are degraded. In an alternative route, the autophagosome can directly fuse with either an endolysosome or a lysosome to become an autolysosome to degrade cargo (Zhao and Zhang, 2019).

The autophagic degradation activity can be measured by the autophagic cargo degradation rate, also known as autophagy flux (Klionsky et al., 2021). During the autophagic degradation process, autophagic cargo and autophagic proteins such as LC3II and autophagy receptors (e.g., p62/SQSTM1) are degraded together. Given this, the protein levels and distribution patterns of LC3II and p62/SQSTM1 are often used to assess the autophagic flux. Thus, an increased level of p62/SQSTM1 immunolabeled intracellular aggregates or LC3-immunopositive puncta on tissue sections often indicates disrupted degradation activity or reduced autophagic flux (Runwal et al., 2019).

### Endolysosomal System

**Figure 1:**
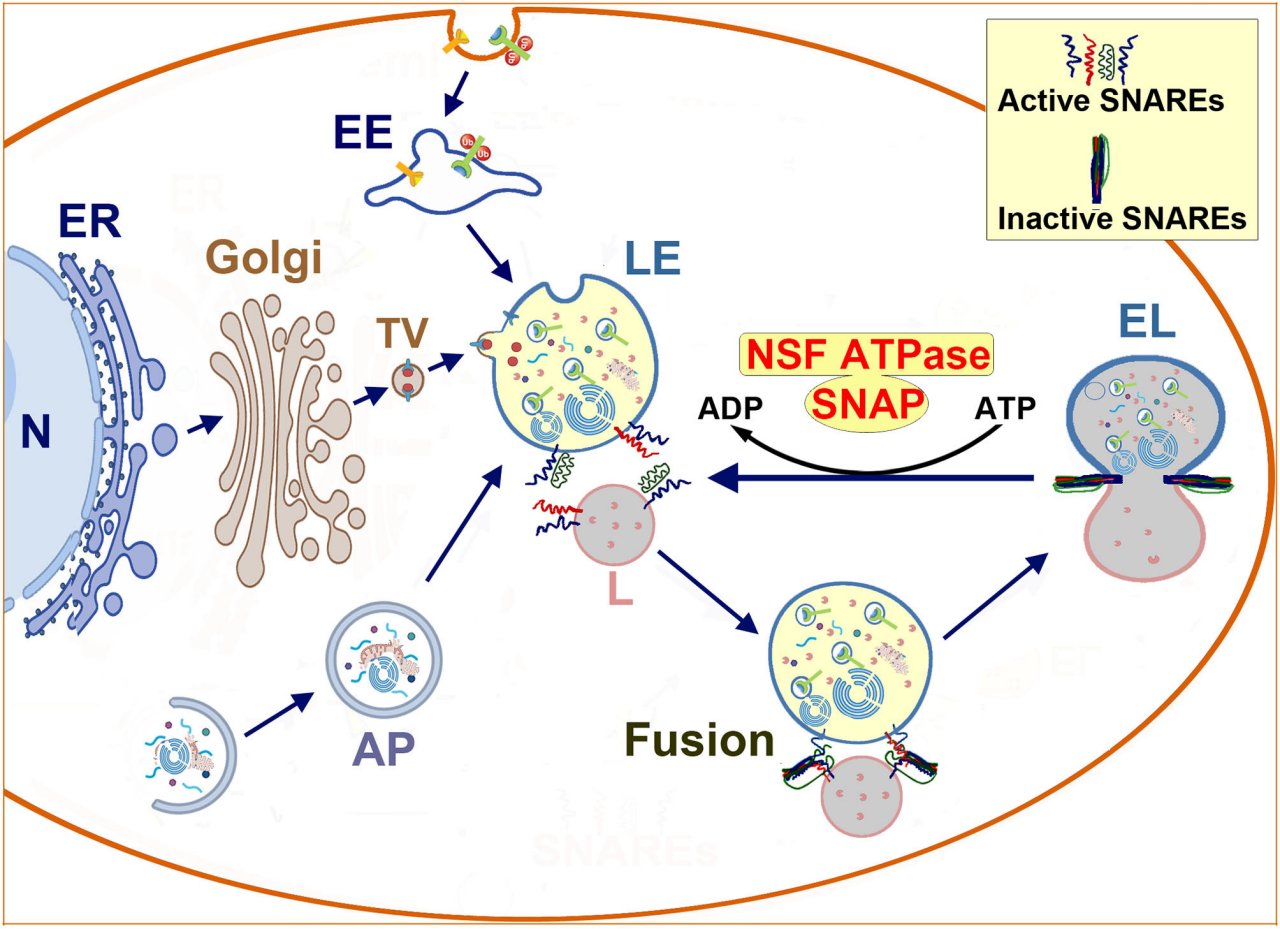
Schematic diagram of endolysosomal trafficking: endolysosomal hydrolytic enzymes, membrane components, and structural proteins are synthesized on the ER-associated polyribosomes, and modified in the ER and the Golgi lumen. Next, they are transported via vesicles to the late endosome. The late endosome also receives incoming cargos from the endocytic and autophagic pathways. The enzyme- and cargo-loaded late endosome fuses with the terminal lysosome to form an endolysosome to digest cargos. Afterwards, the endolysosome reforms into a terminal lysosome for the next round of fusion. The membrane fusion is mediated by the NSF-SNAP-SNAREs machinery and assisted by other proteins such as HOPS complexes and Rab7. Interactions between SNAREs from two opposite lipid membranes bring two membranes together to execute the fusion. After fusion, SNAREs form inactive complexes that must be dissociated and reactivated by the cooperative action of NSF ATPase and SNAP. N, nucleus; ER, endoplasmic reticulum; Golgi, Golgi apparatus; TV, transport vesicle; EE, early endosome; AP, autophagosome; LE, late endosome; L, lysosome; NSF, N-ethylmaleimide Sensitive Factor (ATPase); SNAP, soluble NSF attachment protein; SNARE, SNAP receptor.

As mentioned above, the endolysosomal system is the extension of the trans-Golgi network and the biosynthetic, endocytic, and autophagic pathways. In this review, the endolysosomal system refers to the gamut of organelles, encompassing the late endosome, endolysosome, and terminal lysosome (Figure 1) (Bright et al., 2016; Bissig et al., 2017). Endolysosomal compartments and endolysosomal structures are interchangeable.

There remains some misapprehension of the endolysosomal system. In some studies, the late endosome and endolysosome have been vaguely batched together as the lysosome (Lie and Nixon, 2019). The protein compositions among all three endolysosomal compartments are almost identical, hence this common misconception. Furthermore, because the endolysosomal hydrolytic enzymes and structural proteins are delivered from the trans-Golgi network to the late endosome and then further to endolysosome and lysosome, the late endosome has been considered as the precursor of the endolysosome and lysosome. For that reason, the commonly used lysosomal markers such as cathepsins and lysosomal membrane-associated proteins (LAMPs) are major components of the late endosome, endolysosome, and lysosome. There are no specific protein markers that can discern the late endosome from the endolysosome (Lie and Nixon, 2019). M6PR and Rab7 are the only two proteins currently known to be present in the late endosome and endolysosome, but not in the terminal lysosome (Saftig and Klumperman, 2009). Under transmission electron microscopy (TEM), the late endosome contains ILVs and membrane whorls or fragments, the lysosome appears homogeneous with few membrane fragments, and the endolysosome has morphological characteristics from both the late endosome and terminal lysosome (Tjelle et al., 1996; Huotari and Helenius, 2011). Despite these morphological characteristics, the ultrastructure of the late endosome, endolysosome, and lysosome under EM often remains atypical and thus difficult to differentiate (Griffiths, 1996; Repnik et al., 2013). Another misconception of the lysosome is that they are the major cellular degradation organelle. However, recent studies show that the endolysosome is the principal intracellular site of acid hydrolase activity for degradation of early endosomal and autophagic cargos, whereas the terminal lysosome acts as a store of acid hydrolases and is cathepsin-inactive (Bright et al., 2016).

All endolysosomal compartments have acidic lumens generated by their transmembrane vacuolar adenosine triphosphatase (V-ATPase) (Luzio et al., 2014). The V-ATPase is present throughout the endolysosomal compartments, albeit at different densities, to create a pH range from 6.0 in the late endosome to 4.5 in the lysosome (Yamashiro and Maxfield, 1987). Although the late endosome receives incoming acid hydrolytic enzymes from the Golgi and the degradation cargos from the early endosome and autophagosome, they cannot degrade these waste cargos efficiently because of its higher intraluminal pH (6.0). They must be acidified transiently (“kiss-and-run”) or permanently by fusing with the more acidic terminal lysosome (pH 4.5) to become a hybrid endolysosome (Bright et al., 2016).

After degradation of the cargo, an endolysosome transforms or reforms into a new terminal lysosome. This new terminal lysosome is ready to fuse with a late endosome for the next round of an endolysosome—terminal lysosome cycle (Bright et al., 2016; Bissig et al., 2017; de Araujo et al., 2020). Cells require a dynamic equilibrium of late endosomes, endolysosomes, and terminal lysosomes to maintain homeostasis. An imbalance among endolysosomal compartments leads to a release of their intraluminal hydrolases into the cytoplasm and extracellular space, resulting in cell death (Inpanathan and Botelho, 2019).

The lumens of endolysosomal compartments harbor at least 60 different acid hydrolase enzymes with an optimal pH between 4 and 5, including proteases, nucleases, phosphatases, and lipases. The endolysosomal compartments also contain more than 100 highly glycosylated transmembrane proteins (Xu and Ren, 2015; de Araujo et al., 2020). LAMP1 and LAMP2 are the most abundant transmembrane proteins and make up about 50% of the protein content in the endolysosomal compartment (Luzio et al., 2014). The highly glycosylated transmembrane proteins protect themselves and the lipid membrane from degradation by intraluminal acidic hydrolases. As described in detail in several recent reviews (Condon and Sabatini, 2019; Lawrence and Zoncu, 2019; Ballabio and Bonifacino, 2020), the microphthalmia-associated transcription factor (MiTF)/transcription factor (TF) family, such as transcription factor EB (TFEB), regulates the expression of most endolysosomal proteins. Phosphorylation of TFEB by mammalian target of rapamycin complex 1 (mTORC1) retains TFEB on the endolysosomal outer membranes. To respond to an increase in endolysosomal degradation demand, TFEB is dephosphorylated and translocated to the nucleus to increase the transcriptions of hydrolases (e.g., cathepsins), transmembrane proteins such as LAMPs, and the V-ATPase proton pump complexes.

### Cathepsins

Cathepsins are the most abundant cellular proteases found in the lumen of all three endolysosomal compartments (Yadati et al., 2020). They are classified into three families: serine proteases (A and G), aspartic proteases (D and E), and cysteine cathepsins (B, C, F, H, K, L, O, S, V, X, and W) (Yadati et al., 2020). With a few exceptions, cathepsins show the highest activity in the low pH environment of the endolysosomal lumen. Cathepsins B, L, and D are the most abundant in the endolysosomal compartments (Turk et al., 2000). Cathepsin B (CTSB) is the most abundant cathepsin in the brain (Petanceska et al., 1994). Most cysteine cathepsins exhibit a predominantly endopeptidase activity. Cathepsin B exhibits both exopeptidase and endopeptidase activities favorably at acidic and neutral/alkaline pH values, respectively (Turk et al., 2012). Cathepsin B retains protease activity at the neutral pH found in the extracellular milieu, particularly in the presence of large ECM proteins (Buck et al., 1992; Cavallo-Medved et al., 2011; Turk et al., 2012).

All cathepsins are synthesized as pre-proenzymes on ER-associated polyribosomes. Pre-procathepsins are composed of three segments: a signal peptide, a propeptide, and a mature enzyme. During synthesis, pre-procathepsins are imported into the ER lumen. Inside the ER lumen, pre-procathepsins are cleaved by a signal peptidase to become procathepsins. Procathepsins are then glycosylated with high levels of mannose within the ER before being transported to the Golgi apparatus. In the Golgi, the mannose residues are modified to become mannose-6-phosphate moieties (M6P). The M6P-tagged procathepsins are recognized and bound by M6P-receptors (M6PR), packed together into clathrin-coated vesicles, and transported to the late endosomes (Bright et al., 2016) (Figure 1). Once in the acidic environment of the late endosome, M6PRs release the M6P-procathepsins and then are transported back to the trans-Golgi networks to be reused (Abazeed et al., 2005). Inside the acidic environment (pH 6.0) of the late endosome, the propeptides of procathepsins undergo autocleavage or are cleaved by other cathespins to become mature cathepsins. Cathepsin maturation continues even after the late endosome fuses with a terminal lysosome to become an endolysosome. In the endolysosome, the mature forms of cathepsins are further processed into double heavy chain forms and a light chain. The double heavy chain forms of cathepsins are proteolytically active (Repnik et al., 2013).

### NSF, SNAP, and SNARE-Mediated Membrane Fusion

All eukaryotic cells employ membrane-bound compartments to shuttle macromolecules among organelles or to move them in and out of cells through a process known as membrane trafficking (Yuan et al., 2018a). Figure 1 shows a schematic diagram summarizing the neuronal endolysosomal trafficking pathways and the membrane fusion between a late endosome and a terminal lysosome. The neuronal endolysosomal trafficking pathways include: (i) the delivery of macromolecules through the Golgi to the late endosome; (ii) the endocytic pathway; and (iii) the autophagic pathway. At the trafficking destination, the membrane-bound compartments must fuse to their target membrane for cargo assembly or delivery.

The membrane-to-membrane fusion or simply membrane fusion is the process by which two initially distinct lipid bilayers merge to become a single integrated structure. The membrane fusion is mediated by the cooperative action of several protein complexes and phosphoinositide lipids (Brocker et al., 2010). As shown in Figure 1, the core protein complex is comprised of: (i) N-ethyl-maleimide sensitive factor (NSF), (ii) soluble NSF attachment protein (SNAP), and (iii) soluble NSF attachment protein receptors (SNAREs) (Bombardier and Munson, 2015).

Soluble NSF attachment protein receptors are fusion proteins and can be divided into two categories: vesicle or v-SNAREs, and target or t-SNAREs. Interactions between v-SNAREs and t-SNAREs from two opposite lipid membranes form a trans-SNARE complex, bringing two membranes nearby for their phospholipid membranes to merge into a single organelle (Figure 1). After fusion, SNAREs form an inactive stable cis-complex and must be dissociated by NSF adenosine triphosphatase (ATPase) to become the individual active trans-conformations for the next round of membrane fusion (Baker and Hughson, 2016; Yoon and Munson, 2018). This reactivation requires SNAREs to connect the NSF ATPase mediated by an adaptor protein SNAP (Hong and Lev, 2014) (Figure 1).

N-ethylmaleimide sensitive factor is a homohexamer, with each subunit composed of an amino-terminal domain followed by two AAA+ (ATPases associated with various cellular activities) domains termed D1 and D2. The D2 domain has slow adenosine triphosphate (ATP)-hydrolytic activity to maintain the NSF hexameric structure. The D1 domain is thought to be the main engine that uses ATP hydrolysis for SNARE complex disassembly and reactivation (Yoon and Munson, 2018). There is only a single form of NSF in mammalian cells (Whiteheart et al., 1994). Therefore, deficiency in NSF brings membrane fusion activity to a halt (Wilson et al., 1992; Whiteheart et al., 1994; Mohtashami et al., 2001). In comparison, SNAREs are both abundant and redundant with more than 60 types in yeasts and mammalian cells (Whiteheart et al., 1994). Of the 38 SNAREs present in humans, 30 have been identified in the Golgi and endolysosomal systems (Wilson et al., 1992). The Golgi and endolysosomal compartments contain the highest concentration of NSF and SNAREs, whereas other subcellular organelles such as the ER recruits little NSF (Robinson et al., 1997; Dalal et al., 2004). This distribution suggests that the Golgi and endolysosomal compartments are among the most dynamic organelles with the highest membrane fusion activity (Dingjan et al., 2018).

N-ethylmaleimide sensitive factor-SNAP-SNARE machinery-mediated membrane fusion also requires assistance from several other categories of proteins (Sudhof, 2007). These proteins include coiled-coil tethering complexes, multisubunit tethering complexes, small Rab GTPases, and phosphoinositide lipids (Brocker et al., 2010). In most cases, tethering proteins on opposite membranes connect before v-SNARE and t-SNARE engagement (Pfeffer, 1999; Baker and Hughson, 2016). Examples of these proteins include early endosome antigen 1 (EEA1) which is a coiled-coil membrane tethering protein containing an FYVE domain. The EEA1 FYVE domain binds to phosphatidylinositol-3-phosphate [PtdIns(3)P] on early endosomal membranes (Gaullier et al., 1998). Rab5 is a small GTPase that interacts with EEA1 and is required for the early stages of endosomal membrane-related fusion (Haas et al., 2005). The homotypic fusion and vacuole protein sorting (HOPS) is a multisubunit tethering complex in which complex subunit interactions are required before SNARE-mediated membrane fusion between late endosomes and terminal lysosomes can occur. Rab7 is another small GTPase that interacts with Rab interacting lysosomal protein (RILP) and the HOPS complex during the late endosome and terminal lysosome fusion (Lin et al., 2014).

## Part 2. Neuronal Endolysosomal System

While endolysosomal trafficking has been studied extensively in non-neuronal cells, recent advances have been made in understanding the role of endolysosomal trafficking in highly polarized neurons. Neurons have adapted the same endolysosomal trafficking pathways to accommodate their specific morphological complexity and activity requirements (Winckler et al., 2018). Most neurons require a constant firing of action potentials and a fast turnover of membrane proteins (Harris et al., 2012). The neuronal endolysosomal compartments require extremely high degradative capacities for processing the waste from numerous distal nerve terminals and dendritic branches. These features underscore the need for highly efficient endolysosomal trafficking activities and intricate waste transport systems (Boland et al., 2008; Cai et al., 2010; Lee et al., 2011; Cheng et al., 2015; Gowrishankar et al., 2015; Maday and Holzbaur, 2016; Tammineni and Cai, 2017; Yap et al., 2018; Yuan et al., 2018a).

The extraordinarily high demand of endolysosomal trafficking activities in neurons relative to other cellular systems is supported by emerging gene knockout studies. Knockout of key ATG proteins, ATG5 and ATG7, primarily induces neuronal death (Hara et al., 2006; Komatsu et al., 2006). Rab7 knockout in mice causes an explicit buildup of neuronal late endosomes or autophagosomes, leading to neurodegeneration (Hyttinen et al., 2013).

Sophisticated neuronal waste transport systems are required to cope with the long-distance trafficking from distal synaptic connections to the soma region where the endolysosomal degradation compartments are located (Maday et al., 2012; Cheng et al., 2015; Maday and Holzbaur, 2016; Giovedi et al., 2020). Treatment with bafilomycin A1, which targets the V-ATPase, blocks the acidification of the endolysosomal compartments, resulting in the accumulation of autophagosomes exclusively within the soma region. This result supports the concept that the neuronal soma region is the primary site of cargo degradation (Maday and Holzbaur, 2016). As demonstrated below, brain ischemia also leads to a buildup of autophagosomes and endosomes within the soma region, but not in the neuropil area where distal neuronal processes and synapses are distributed (see **Figures 3–6**).

A dysfunctional endolysosomal system has been observed across a broad spectrum of neurodegenerative disorders, including Alzheimer's disease, Parkinson's disease, Huntington's disease, and amyotrophic lateral sclerosis (Winckler et al., 2018; Lie and Nixon, 2019; Giovedi et al., 2020; Navarro-Romero et al., 2020; Qureshi et al., 2020). Lysosomal storage disorders (LSDs) favorably affect neurons as well (Navarro-Romero et al., 2020; Qureshi et al., 2020; Song et al., 2020). Recent studies also show that stroke brain injury results from damage to endolysosomal trafficking (Yuan et al., 2018a). These studies suggest that dysfunction of endolysosomal trafficking may play a key role in most, if not all, neuronal death processes, although the mechanism underlying the deficiencies varies among neurological disorders.

## Part 3. Interruption of Endolysosomal Trafficking Leads to Stroke Brain Injury

Alteplase (tPA) and endovascular treatment may improve clinical outcomes. However, only 5–10% of stroke patients are eligible for these reperfusion therapies. Furthermore, these reperfusion therapies rely on removing vascular blockage and can inadvertently lead to an ischemia-reperfusion injury. At present, there exist no therapies directed toward brain ischemia-reperfusion injuries.

Smith et al. (1984) found that 10 min of global brain ischemia in a rat model led to selective and delayed neuronal death in some populations of hippocampal, striatal, and neocortical neurons at 2–3 days after reperfusion. Compared to the brief episode of global brain ischemia (prolonged) focal brain ischemia (stroke) with or without reperfusion leads to a well-described progression from early infarction in the striatum to delayed infarction in the dorsolateral cortex overlying the striatum (Barber et al., 2004; Carmichael, 2005). About 80% of neurons die in the non-perfused core with complete ischemia within 4 h, whereas about 40% of neurons gradually die in the hypoperfused penumbral region over a period of 4–24 h after focal brain ischemia (Fifield and Vanderluit, 2020). The breakdown of the blood brain barrier, microglia activation, monocyte/neutrophil infiltration, and astrogliosis were just observed until 24 h after focal brain ischemia (Fifield and Vanderluit, 2020). In general, animals cannot survive a global brain ischemic episode lasting longer than 25–30 min. A brief episode of global brain ischemia is mostly used to model the brain ischemia after a transient cardiac arrest in humans (Yuan et al., 2018a). Alternatively, animals can survive prolonged focal brain ischemia with or without reperfusion. Focal brain ischemia in animals is exclusively used to model an ischemic stroke in humans (Hu et al., 2001).

Deficiency in the endolysosomal system is observed virtually in all neurodegenerative disorders and in a significant number of LSDs (Wang et al., 2018; Winckler et al., 2018; Lie and Nixon, 2019; de Araujo et al., 2020; Giovedi et al., 2020; Navarro-Romero et al., 2020; Qureshi et al., 2020). Recent studies show that interruption of endolysosomal trafficking also leads to post-stroke brain ischemia-reperfusion injury. The interruption of endolysosomal trafficking in post-stroke neurons leads to: (i) the complete fragmentation of the Golgi apparatus; (ii) a massive buildup of enlarged endolysosomal compartments filled with undigested material; (iii) a massive increase in autophagosomes due to the blockade of autophagic flux; (iv) a massive accumulation of enlarged early endosomal structures due to the blockade of the late endosomal fusion; and (v) a significant buildup of protein aggregates (Hu et al., 2000b, 2001; Yuan et al., 2018a,b).

**Figure 2:**
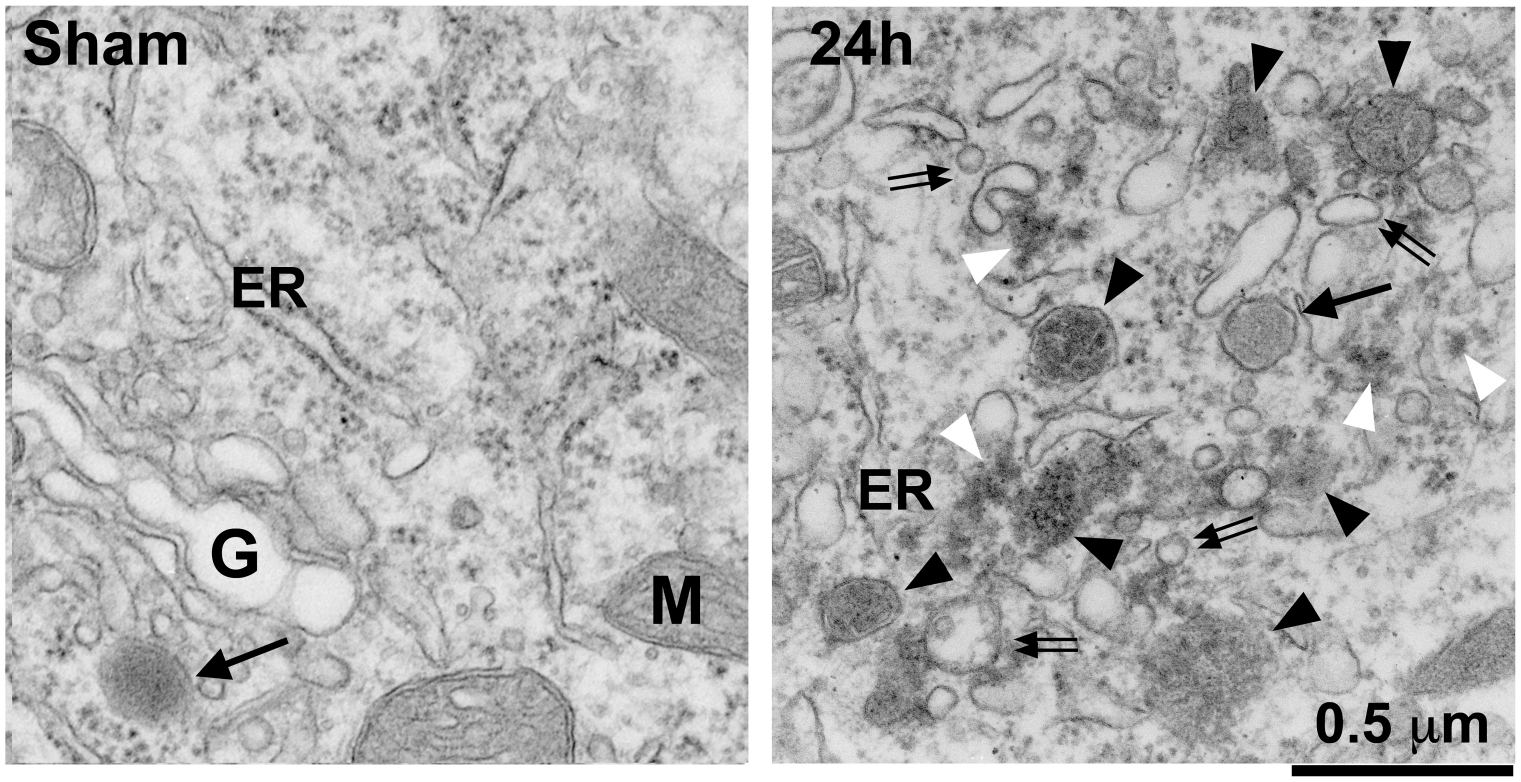
Electron microscopy of Golgi fragmentation and buildup of endolysosomal structures and protein aggregates after stroke. The ultrathin neocortical sections were obtained from a sham-operated control rat and a rat subjected to 2 h of MCAO followed by 24 h of reperfusion. ***Sham:*** A sham neuron showing normal rough endoplasmic reticulum (ER), mitochondria (M), Golgi apparatus (G), and lysosome (arrow). ***24 h:*** A 24 h reperfused neuron showing complete fragmentation of the Golgi apparatus into vesicles or vacuoles (double arrows) and buildup of various sized late endosomes (black arrowheads) and protein aggregates (white arrowheads). Arrow points to a terminal lysosome. Scale bar = 0.5 μm.

Figure 2 shows that, under TEM, complete fragmentation of the Golgi apparatus and a massive accumulation of protein aggregates and endolysosomal structures are observed in these post-ischemic neurons. These protein aggregates and endolysosomal structures become the foremost intra-neuronal structures after both global (Hu et al., 2000b; Liu and Hu, 2004) and focal brain ischemia (Hu et al., 2001; Zhang et al., 2006) (Figure 2).

### Inactivation of NSF Leads to Interruption of Neuronal Endolysosomal Trafficking After Brain Ischemia

As described in Part 1 of this review, the NSF-SNAP-SNARE machinery is the prerequisite for membrane fusion (see Figure 1). Interactions between v-SNAREs and t-SNAREs from two opposite lipid membranes execute membrane fusion (Yoon and Munson, 2018). After fusion, SNAREs form inactive stable cis-complexes, which must be dissociated and then reactivated by NSF for the next round of membrane fusion (Baker and Hughson, 2016; Yoon and Munson, 2018). There is only a single form of NSF in mammalian cells (Whiteheart et al., 1994). Therefore, deficiency in NSF brings membrane fusion activities to a halt (Wilson et al., 1992; Whiteheart et al., 1994; Mohtashami et al., 2001; Yuan et al., 2018a,b). This halt on membrane fusion activity handicaps the endolysosomal trafficking system from performing its job leading to neuronal dysfunction and injury.

There are a few *in vivo* studies referencing the NSF-SNAP-SNARE machinery after brain ischemia. Most of these studies are reported by our group. Liu et al. (Liu and Hu, 2004) found that functional NSF was depleted because it was deposited into inactive Triton X100-insoluble protein aggregates as early as after 30 min of reperfusion in the hippocampal CA1 neurons. This persisted until neuronal death occurred at 2–3 days of reperfusion following the initial ischemic episode. In comparison, NSF remained active in the dentate gyrus neurons that survived the same ischemic episode. The inactive deposition of NSF was accompanied by complete fragmentation of the Golgi stacks and accumulation of large quantities of intracellular vesicular structures (mainly due to Golgi fragmentation) in hippocampal CA1 neurons. This study showed that permanent depletion of active NSF likely led to delayed neuronal death in CA1 neurons even after just a brief episode of global brain ischemia.

Yuan et al. (2018b) found that 20 min of global brain ischemia led to the selective and complete inactivation of NSF ATPase in post-ischemic neurons. They discovered that NSF inactivation leads to a massive accumulation of Golgi fragments, intracellular vesicles, and cargo-laden late endosomes in affected post-ischemic neurons. They also found that the presence of 33 kDa CTSB was significantly increased in the cytosolic fraction likely released from late endosomes, and these neurons with increased cytosolic CTSB underwent delayed neuronal death. However, CTSB release was mostly limited inside neurons, suggesting that the release was likely on a microscale level. They suggested that the microscale release of cathepsins might not cause catastrophic destruction of neuronal structures or tissue infarction indiscriminately; instead, they might specifically activate the truncated Bax-like BH3 protein (tBid) and BCL2-associated X protein (Bax) pathway. As a result, neuronal mitochondrial outer membrane permeabilization (MOMP) occurs, leading to delayed neuronal death after a brief episode of global brain ischemia (Yuan et al., 2018a).

Recent work from our laboratory showed that (prolonged) focal brain ischemia caused a large-scale release of CTSB that damaged all neuronal structures indiscriminately. As a result, the post-ischemic neurons rupture, releasing their contents which include CTSB, ubiquitinated protein aggregates, and p62-protein aggregates into the extracellular space.

**Figure 3:**
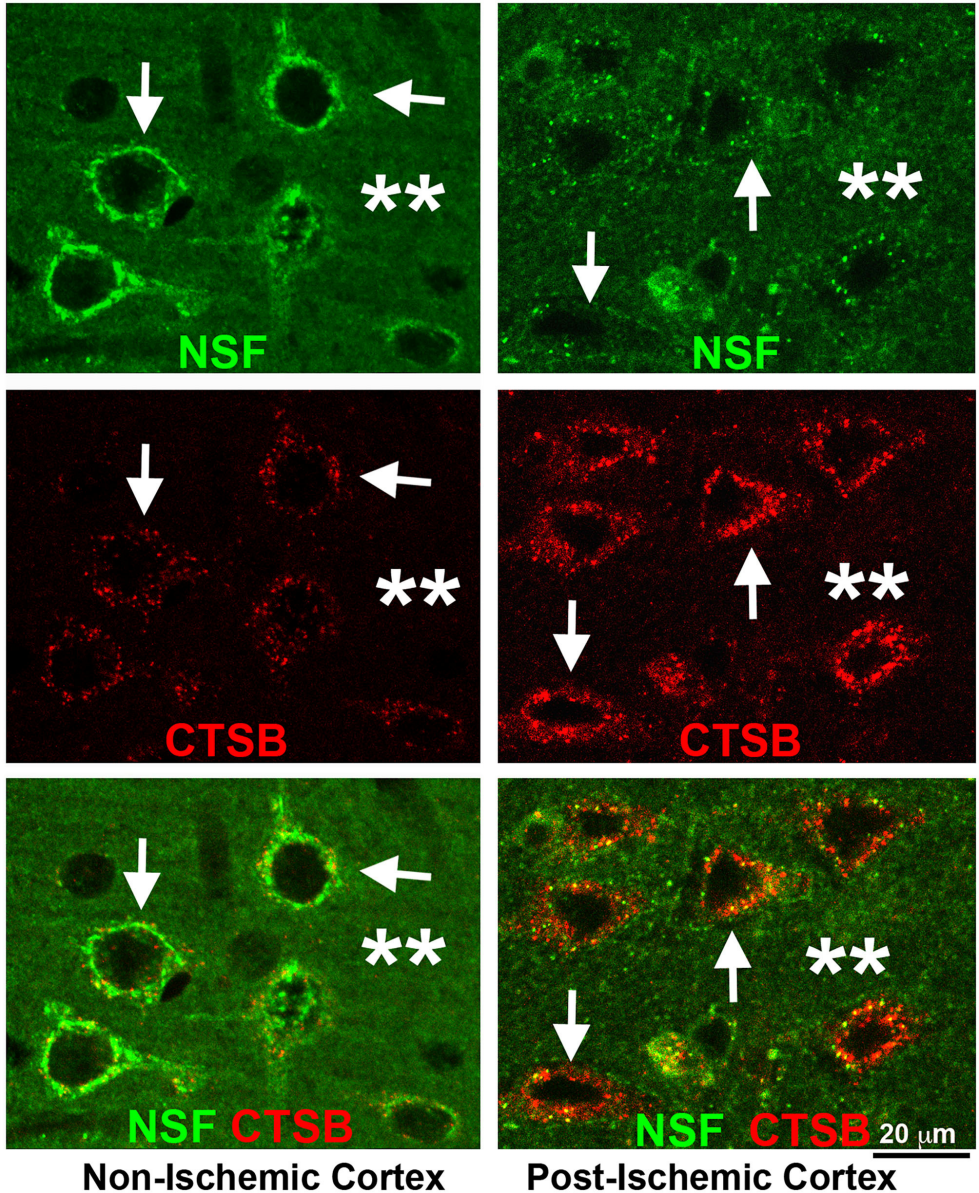
Confocal imaging of neuronal NSF depletion and CTSB buildup after stroke. Brain section obtained from a rat subjected to 2 h of MCAO followed by 24 h of reperfusion, double-labeled with NSF (green) and CTSB (red) antibodies, and examined by confocal microscopy. Left panels display the non-ischemic contralateral neocortex; Right panels show the post-ischemic ipsilateral neocortex. **Upper row:** NSF immunolabeling; **Middle row:** CTSB immunolabeling; **Lower row:** NSF (green) and CTSB (red) double-immunolabeling. Arrows point to neuronal soma; and stars denote the immunostaining outside neuronal soma.

Yuan et al. (2021) showed that NSF was irreversibly depleted or inactively deposited in virtually all penumbral neurons after 2 h of middle cerebral artery occlusion (MCAO) in the rat model. Figure 3 shows that NSF was irreversibly depleted from virtually all penumbral neurons after 2 h of MCAO in the rat model. Brain sections of 2 h of MCAO followed by 24 h of reperfusion were double-immunolabeled with mouse anti-CTSB (red) and rabbit anti-NSF (green) antibodies (Figure 3). Both CTSB and NSF immunostainings were predominantly distributed in neurons (Figure 3, arrows). In the non-ischemic contralateral cortex, anti-NSF immunoreactivity was distributed in the perinuclear region and apical dendritic trunk (Figure 3, Non-Ischemic Cortex, upper left, NSF, green, arrows), as well as in the neuropil region (Figure 3, Non-Ischemic Cortex, upper left, NSF, green, stars). Anti-CTSB antibody labeled the endolysosomal structures as small dots in the same perinuclear region and apical dendritic trunk (Figure 3, middle left, CTSB, red, arrows), but CTSB immunolabeling was virtually absent in the neuropil or extracellular space between neuronal soma of the non-ischemic contralateral cortex (Figure 3, middle left, CTSB, red, stars). Furthermore, there was little colocalization between NSF (green) and CTSB (red) immunoreactivities in the non-ischemic contralateral neocortical neurons (Figure 3, lower left, green vs. red, arrows). In contrast, in the post-ischemic ipsilateral cortex, NSF immunostaining was mostly depleted from inside the neuronal soma and dendritic trunks, only leaving a few NSF-immunopositive small dots in the perinuclear area (Figure 3, Post-ischemic cortex, upper right, NSF, green, arrows). In the same post-MCAO penumbral neurons, the size, intensity, and number of CTSB-immunostained endolysosomal structures were drastically increased (Figure 3, Post-ischemic cortex, middle right, CTSB, red, arrows). Cathepsin B-immunostaining was also distributed outside neuronal soma (Figure 3, Post-ischemic cortex, middle right, CTSB, red, stars), indicating that some damaged neurons had ruptured and released their contents including CTSB into the extracellular space. Furthermore, NSF (green) and CTSB (red) appeared partially colocalized in the perinuclear region of the penumbral neurons (Figure 3, lower right, yellow, small arrows). These results suggest that NSF ATPase was irreversibly depleted virtually from all penumbral neurons, resulting in a massive buildup of CTSB-containing endolysosomal structures. Penumbral neurons then rupture and release their contents into the extracellular space, causing catastrophic destruction of neuronal structures indiscriminately.

### Mechanism Underlying Post-Ischemic NSF Inactivation

As described above, the depletion or disappearance of intra-neuronal NSF immunoreactivity was mainly due to NSF deposition into a detergent (Triton X100)/salt-insoluble aggregates after both global and focal brain ischemia (Liu and Hu, 2004; Yuan et al., 2018b, 2021; Liu et al., 2021). These NSF aggregates are such dense structures that they were not accessible by the NSF antibody during the immunostaining of brain sections (Hu et al., 2000b; Liu and Hu, 2004; Zhang et al., 2006; Yuan et al., 2018b, 2021).

N-ethylmaleimide sensitive factor (ATPase) requires binding to ATP or non-hydrolysable ATP analogs to maintain its active soluble symmetrical homohexamer conformation (Moeller et al., 2012). All previous studies of NSF activity have consistently shown that the cytosolic free NSF is in its active form, whereas NSF deposition into Triton X100-insoluble aggregates loses its ATPase activity (Mohtashami et al., 2001; Sanyal and Krishnan, 2001). Cellular NSF forms a symmetrical homohexamer that undergoes drastic conformational change from an ATP- to a non-ATP-binding state (Yu et al., 1998; Moeller et al., 2012). N-ethylmaleimide sensitive factor requires ATP for stability (Moeller et al., 2012; Morgan and Burgoyne, 2015). Without ATP as a cofactor, NSF undergoes inactive aggregation in nucleotide-free solutions (Block et al., 1988; Whiteheart et al., 1992; Moeller et al., 2012; Morgan and Burgoyne, 2015). The underlying mechanism for NSF inactive deposition is likely due to an NSF ATPase conformational change from a compact cylindrical structure to an “aggregation-prone” open structure under ATP depleted conditions (Hanson et al., 1997; Yu et al., 1998; Moeller et al., 2012). Adenosine triphosphate is depleted within a couple of minutes following brain ischemia (Siesjo et al., 1999). Therefore, ATP depletion after brain ischemia leads to the inactive deposition of NSF hexamer ATPase into Triton X100-insoluble aggregates as described above (Hu et al., 2000b; Liu and Hu, 2004; Zhang et al., 2006; Yuan et al., 2018b, 2021). N-ethylmaleimide sensitive factor also formed inactive aggregates due to a conformational change in tissue samples from mice with genetically induced leucine-rich repeat kinase 2 (LRRK2)-deficiency in a Parkinson's disease mouse model (Pischedda et al., 2021). N-ethylmaleimide sensitive factor is persistently deposited in the Triton X100-insoluble aggregate form until neuronal death occurs during the post-ischemic phase, suggesting that post-ischemic NSF aggregation is an irreversible process (Yuan et al., 2018b, 2021).

### Complete Fragmentation of Golgi Complex After Focal Brain Ischemia

The Golgi apparatus, also known as the Golgi complex or simply the Golgi, processes and packages proteins into membrane-bound transport vesicles destined for the secretory and endocytic pathways. The Golgi is organized into the first cisterna called cis-Golgi network, medial cisternae, and the final cisterna known as the trans-Golgi network. The cis-Golgi network receives incoming proteins from transport vesicles derived from the ER. The proteins are further modified in the cis-Golgi network, middle cisternae, and trans-Golgi network. These proteins are then packed into transport vesicles at the trans-Golgi network and relayed to their final destinations.

The fragmentation of the Golgi stacks and the buildup of membranous whorls were first observed under EM more than three decades ago from brain tissue after a brief episode of global brain ischemia (Petito and Pulsinelli, 1984; Rafols et al., 1995; Hu et al., 2000a). We now know that these cytoplasmic membranous whorls likely represent the significant buildup of cargo-laden endolysosomal structures (Hall et al., 1997; Hu et al., 2000a, 2001; Yuan et al., 2018a). Despite this knowledge, only a few direct studies focus on the Golgi's morphological and functional characteristics after focal brain ischemia in animal stroke models. A recent study from our laboratory demonstrated that the Golgi apparatus was severely disintegrated and eventually dissolved in post-ischemic neurons destined for death after 2 h of MCAO in a rat model (Yuan et al., 2021).

**Figure 4:**
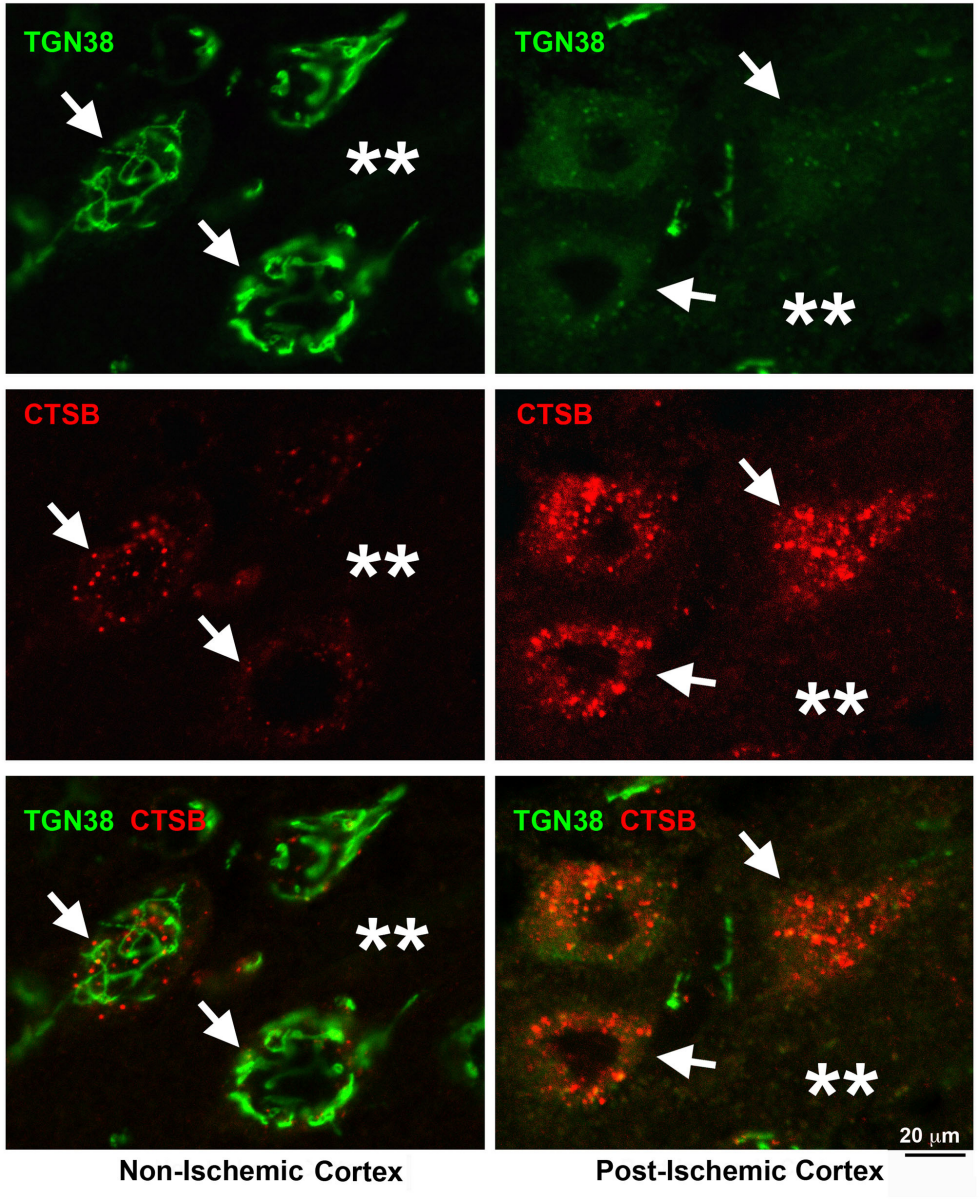
Confocal imaging of neuronal Golgi fragmentation after stroke. Brain section obtained from a rat subjected to 2 h of MCAO followed by 24 h of reperfusion, double-labeled with TGN38 (green) and CTSB (red) antibodies, and examined by confocal microscopy. Left panels display the contralateral non-ischemic cortex; Right panels show the ipsilateral post-ischemic cortex. **Upper row**: TGN38 immunolabeling; **Middle row**: CTSB immunolabeling; **Lower row:** TGN38 (green) and CTSB (red) double-immunolabeling. Arrows point to neuronal soma; and stars denote the immunostaining outside neuronal soma.

Figure 4 shows that the Golgi networks disintegrated into small dots and dissolved into diffuse immunostainings while the cytoplasmic endolysosomal structures markedly accumulate in penumbral neurons after MCAO. Brain sections from a rat subjected to 2 h of MCAO followed by 24 h of reperfusion were double-immunolabeled with TGN protein 38 (TGN38, a Golgi marker) and CTSB antibodies (Figure 4). In non-ischemic contralateral neocortical neurons, TGN38 immunoreactivity was distributed as twisted tubular networks (Figure 4, Non-ischemic cortex, upper left, TGN38, green, arrows) while CTSB immunoreactivity showed as tiny dots in the perinuclear region and apical dendrites (Figure 4, Non-ischemic cortex, middle left, CTSB, red, arrows). There was little overlap between TGN38 and CTSB (Figure 4, Non-ischemic cortex, lower left, green vs. red, arrows). In contrast, TGN38 disintegrates at 24 h of reperfusion following 2 h of MCAO (Figure 4, Post-ischemic cortex, upper right, TGN38, green, arrows), while CTSB increased in size, intensity, and number (Figure 4, Post-ischemic cortex, middle right, CTSB, red, arrows). Furthermore, TGN38 appeared to be overlapping with the CTSB structures in post-ischemic neurons (Figure 4, Post-ischemic cortex, lower right, arrows). Post-ischemic neurons became polygonal in shape and were surrounded with higher extracellular levels of TGN38 and CTSB immunoreactivities (Figure 4, Post-ischemic cortex, lower right, stars), suggesting that some penumbral neurons ruptured, releasing their contents into the extracellular space.

Evidence suggests that the Golgi cisterns irreversibly disintegrate and eventually dissolve prior to penumbral neurons' rupture after 2 h of focal brain ischemia. As a result, the transport of hydrolytic enzymes and structural proteins from the trans-Golgi network to the late endosome is impeded, retaining CTSB in the fragmented Golgi and contributing to the endolysosomal deficiency. Furthermore, the Golgi structural damage releases toxic CTSB into the cytoplasm and extracellular space, increasing stroke brain injury.

### Interruption of Autophagic Flux After Brain Ischemia

Autophagy is a major degradation pathway responsible for removing abnormal protein aggregates and damaged organelles (Liu et al., 2010). Increases in autophagic components, such as LC3-II, beclin-1, TFEB, and aggregated p62/SQSTM1 were consistently observed in neurons of various brain ischemia and hypoxia animal models (Nitatori et al., 1995; Adhami et al., 2007; Carloni et al., 2008; Rami et al., 2008; Wen et al., 2008; Liu et al., 2010, 2018).

As mentioned above, the most common methods for measuring the autophagic cargo degradation rate or autophagic flux employ a combination of techniques including Western blotting and confocal or fluorescence microscopic analysis. These methods measure the levels and immunolabeling patterns of autophagosome-associated proteins, such as LC3-II and autophagy receptors (e.g., p62/SQSTM1). The increased levels of LC3-II and p62/SQSTM1 often indicate that autophagic flux is disrupted or autophagic degradation activity is reduced (Runwal et al., 2019). Autophagic flux can also be measured by EM (Liu et al., 2010; du Toit et al., 2018). Electron microscopy has been considered to be the gold standard in many autophagy research applications because it has the advantage of allowing a direct assessment of autophagosomes in the specimen (Liu et al., 2010; du Toit et al., 2018). Electron microscopy manifestation of the accumulation of mature autophagosomes located in close proximity to enlarged endolysosomal compartments often indicates that the autophagic flux is interrupted, mostly owing to the deficiency in the fusion and degradation steps of the autophagic pathway (Abada et al., 2017). Although EM has an advantage in viewing endolysosomal structures, relative to Western blotting and confocal microscopy, EM methods require multiple steps of sample preparation and specific EM equipment (Liu et al., 2010).

Presently, there are two opposing hypotheses in literature stating that post-ischemic autophagy either increases or decreases brain ischemia reperfusion injury. However, there is no unified theory to reconcile these two opposing hypotheses. In the autophagic pathway, autophagosomes must fuse with endolysosomal compartments for degradation of the autophagic cargos. This fusion is mediated by the NSF-SNAP-SNARE machinery (Abada et al., 2017). Therefore, depletion of NSF in post-ischemic neurons predictably leads to the blockade of autophagic flux at the fusion step between autophagosomes and the endolysosomal compartments.

**Figure 5:**
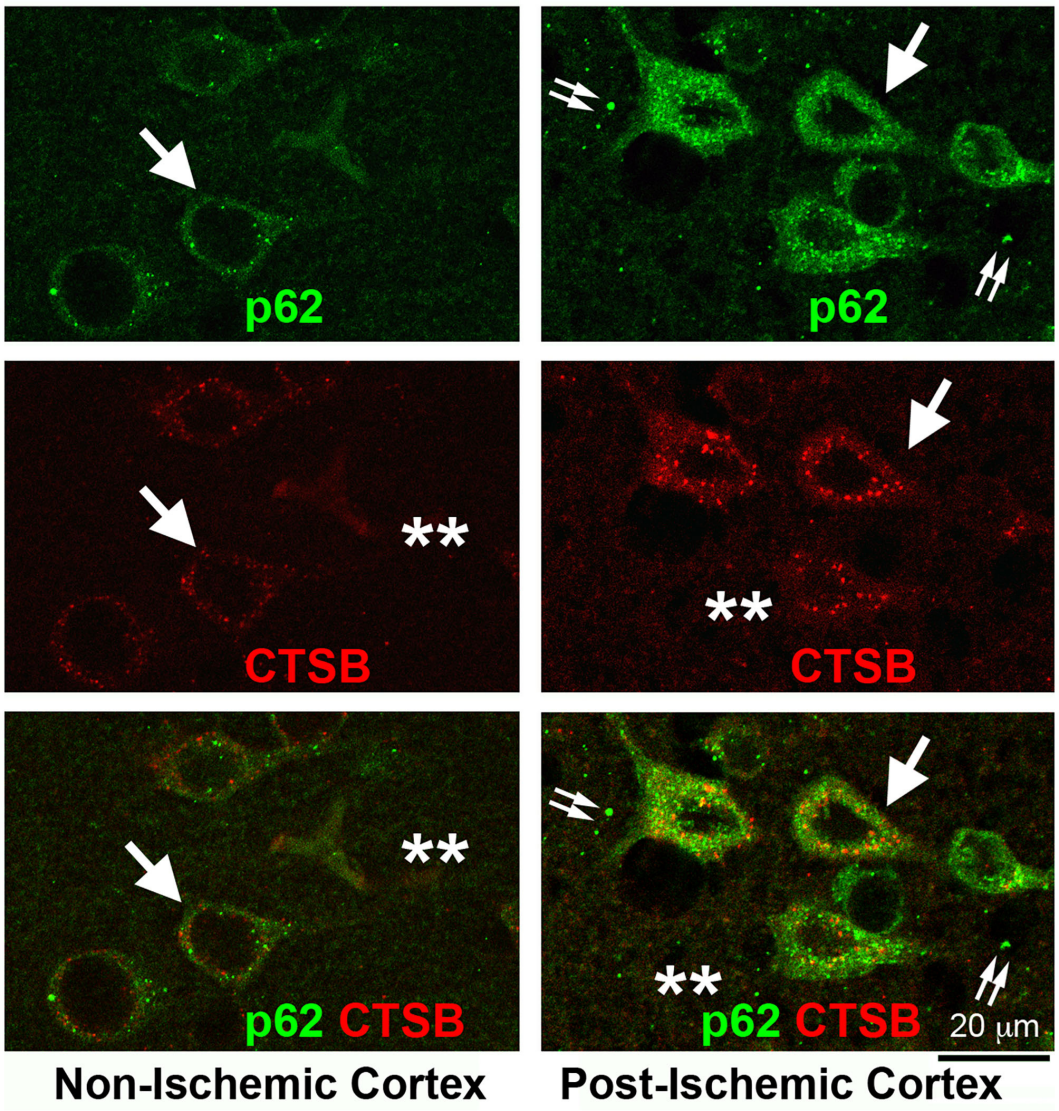
Confocal imaging of neuronal buildup of p62/SQSTM1 and CTSB after stroke. Brain section obtained from a rat subjected to 2 h of MCAO followed by 24 h of reperfusion, double-labeled with p62/SQSTM1 (green) and CTSB (red) antibodies, and examined by confocal microscopy. Left panels display the contralateral non-ischemic cortex; Right panels show the ipsilateral post-ischemic cortex. **Upper row:** p62/SQSTM1 immunolabeling; **Middle row:** CTSB immunolabeling; **Lower row**: p62/SQSTM1 (green) and CTSB (red) double-immunolabeling. Arrows point to p62/SQSTM1 and CTSB immunolabeling in neuronal soma; double-arrows indicate the p62/SQSTM1 immunolabeling outside of neuronal soma; and stars denote the immunostaining outside of neuronal soma.

This NSF-depletion hypothesis is supported by recent work from our laboratory. Figure 5 shows the massive buildup of p62/SQSTM1 immunostained structures in post-ischemic neurons and the nearby extracellular space after focal brain ischemia. Brain sections from a rat subjected to 2 h of MCAO followed by 24 h of reperfusion were double-immunolabeled with p62/SQSTM1 and CTSB antibodies. p62/SQSTM1 immunoreactivity was evenly distributed and occasionally seen as small dots in non-ischemic contralateral neocortical neurons (Figure 5, Non-ischemic cortex, upper left, p62, green, arrows), while CTSB immunolabeled as small dots and was primarily distributed in the perinuclear and apical dendritic regions (Figure 5, Non-ischemic cortex, middle left, CTSB, red, arrows). There was little immunostained CTSB in the neuropil and extracellular space (Figure 5, Non-ischemic cortex, middle left, CTSB, red, stars) and virtually no overlap between p62/SQSTM1 and CTSB immunoreactivities (Figure 5, Non-ischemic cortex, lower left, green vs. red, arrows). In contrast, in the post-ischemic ipsilateral penumbral neocortical neurons, both p62/SQSTM1 (Figure 5, Post-ischemic cortex, upper right, p62, green, arrows) and CTSB (Figure 5, Post-ischemic cortex, middle right, CTSB, red, arrows) immunoreactivities were markedly upregulated in size, number, and intensity. Furthermore, both p62/SQSTM1 and CTSB immunoreactivities appeared also in the neuropil and extracellular space (Figure 5, Post-ischemic cortex, upper and middle right, double-arrows, and stars). There remained little colocalization between p62/SQSTM1 and CTSB both inside and outside of post-ischemic penumbral neuronal soma (Figure 5, lower right, p62 and CTSB, green vs. red, arrows, double-arrows, and stars). These results strongly suggest that the accumulation of p62/SQSTM1-immunostained autophagosomes was located in close proximity to the CTSB-positive endolysosomal compartments but that they were not fused together after focal brain ischemia. Evidence demonstrates that the autophagic flux is interrupted because of deficient fusion between autophagosomes and endolysosomal compartments owing to NSF depletion in post-ischemic neurons.

### Buildup of EEA1 Endosomal Structures After Brain Ischemia

There are only a few studies of the endocytic pathway after brain ischemia. (Vaslin et al., 2009, 2011) showed that all tracers were taken up by endocytosis selectively into post-ischemic neurons destined for death in a rat focal brain ischemia model. The endocytosed tracers were colocalized with enhanced immunolabeling for EEA1 and clathrin in the same population of penumbral neurons after focal brain ischemia. The results suggest a drastic endosomal structure buildup after focal brain ischemia (Vaslin et al., 2009). Huang et al. (2013) showed an accumulation of internalized aquaporin 4 (AQP4) in the increased EEA1-immunolabeled structures at 1 h and then accumulation in the LAMP1-immunolabeled structures at 3 h after focal brain ischemia in the rat model. This finding suggested that AQP4 was internalized and delivered from the EEA1-immunopositive early endosome to the LAMP1-immunopositive endolysosomal compartments after brain ischemia. These studies demonstrated a buildup of early (EEA1-immunopositive) and late (LAMP1-immunopositive) endosomal structures that occur in post-ischemic neurons destined for death after focal brain ischemia. However, similar to the increased autophagosomal structures, the increase in cargo-carrying endosomal structures may not only suggest boosted endocytic activity, but also more likely an indication of the reduced degradation of endocytosed structures in post-ischemic neurons.

**Figure 6:**
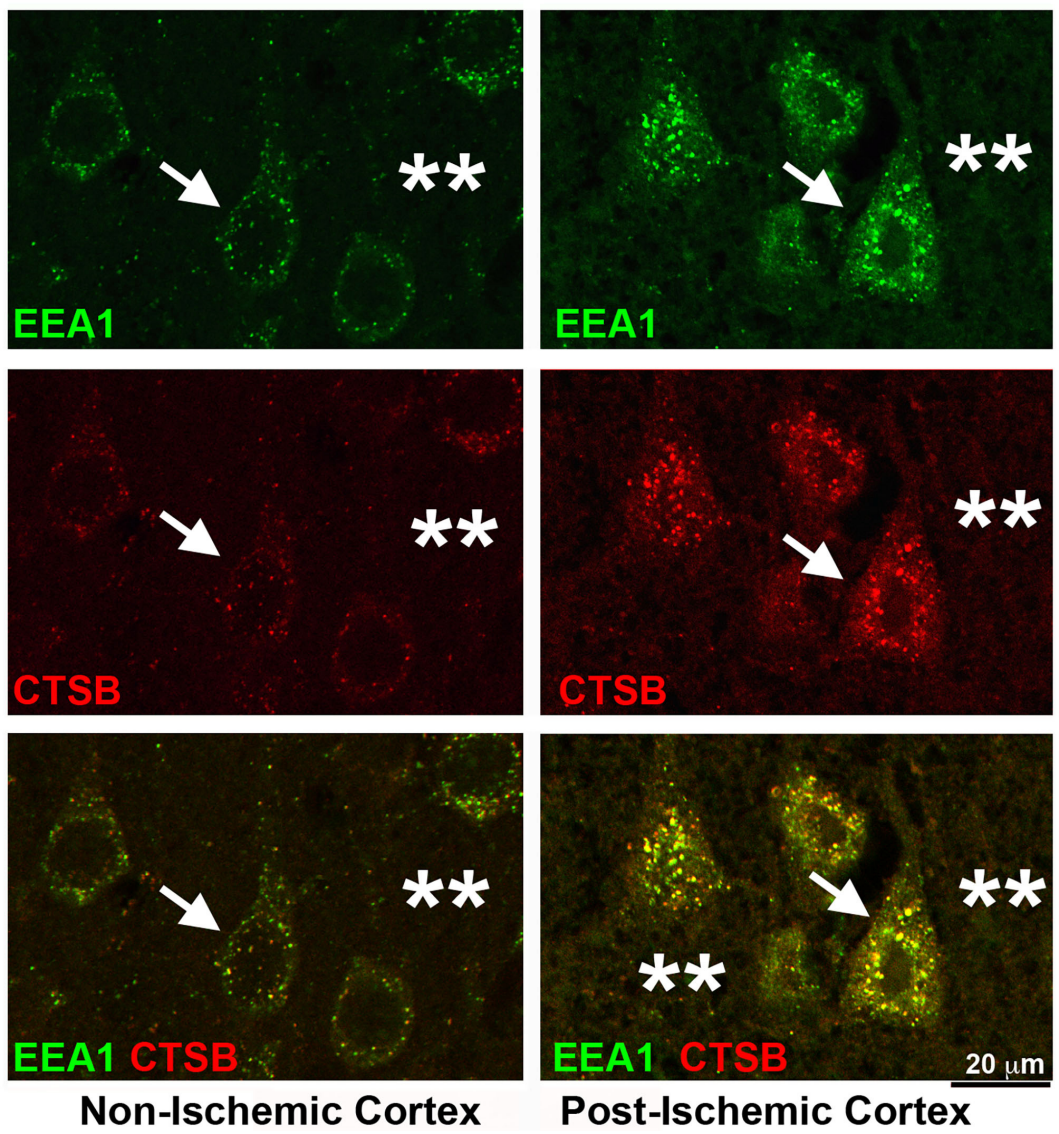
Confocal imaging of neuronal buildup of EEA1 and CTSB after stroke. Brain section obtained from a rat subjected to 2 h of MCAO followed by 24 h of reperfusion, double-labeled with EEA1 (green) and CTSB (red) antibodies and examined by confocal microscopy. Left panels display the contralateral non-ischemic cortex; Right panels show the ipsilateral post-ischemic cortex. **Upper row**: EEA1 immunolabeling; **Middle row**: CTSB immunolabeling; **Lower row**: EEA1 (green) and CTSB (red) double-immunolabeling. Arrows point to neuronal soma; and stars denote the immunostaining outside neuronal soma.

Depletion of NSF blocks the fusion between the late endosomes and terminal lysosomes. As a result, the endosomal structures accumulate after focal brain ischemia. Figure 6 shows that immunoreactivities of both EEA1 and CTSB were drastically increased in penumbral neurons after focal brain ischemia. Brain sections from a rat subjected to 2 h of MCAO followed by 24 h of reperfusion were double-immunolabeled with EEA1 and CTSB antibodies. In the non-ischemic contralateral neocortex, EEA1 immunoreactivity was distributed as small dots throughout the soma, dendritic trunks, as well as, to a significant lesser degree, the neuropil region (Figure 6, upper left, EEA1, green, arrow, and stars), while CTSB immunoreactivity was limited to only the perinuclear and apical dendritic regions (Figure 6, Non-Ischemic Cortex, middle left, CTSB, red, arrow). There were partial colocalizations between EEA1 and CTSB immunoreactivities in non-ischemic contralateral neocortical neurons (Figure 6, Non-Ischemic Cortex, lower left, green vs. red, arrow). In contrast, in post-ischemic ipsilateral penumbral neocortical neurons, both EEA1 (Figure 6, Post-Ischemic Cortex, upper right, EEA1, green, arrows) and CTSB (Figure 6, middle right, CTSB, red, arrows) immunoreactivities were markedly increased in size, number, and intensity in the perinuclear region. The EEA1 and CTSB immunoreactivities were mostly colocalized in the perinuclear region of post-ischemic penumbral neurons (Figure 6, Post-Ischemic Cortex, lower right, EEA1, and CTSB, green vs. red, arrows). The EEA1 and CTSB immunoreactivities were also found in the extracellular space (Figure 6, right panels, stars). These results suggest that increased EEA1-immunopositive early endosomes matured to become the CTSB-immunopositive late endosomes but were not able to fuse with terminal lysosomes owing to NSF depletion in post-ischemic neurons.

### NSF Inactivation Is Responsible for the Golgi, Endosomal, and Autophagic Defects

The following evidence supports the hypothesis that NSF inactivation is responsible for the Golgi, endosomal, and autophagic defects observed after brain ischemia. N-ethylmaleimide sensitive factor was originally identified by Rothman's laboratory based on an assay of Golgi fragmentation in a cell free system (Block et al., 1988). Treatment of the cell free system with alkylating agent N-ethylmaleimide (NEM) leads to Golgi fragmentation. The Golgi fragmentation can then be rescued by adding back an appropriately prepared cytosol fraction. The protein in the cytosol fraction responsible for the rescue was termed NEM-sensitive factor (NSF). For their discoveries of NSF-SNAP-SNARE machinery regulating vesicle traffic, the Nobel Prize in Physiology or Medicine 2013 was awarded jointly to James E. Rothman, Randy W. Schekman, and Thomas C. Südhof.

**Figure 7:**
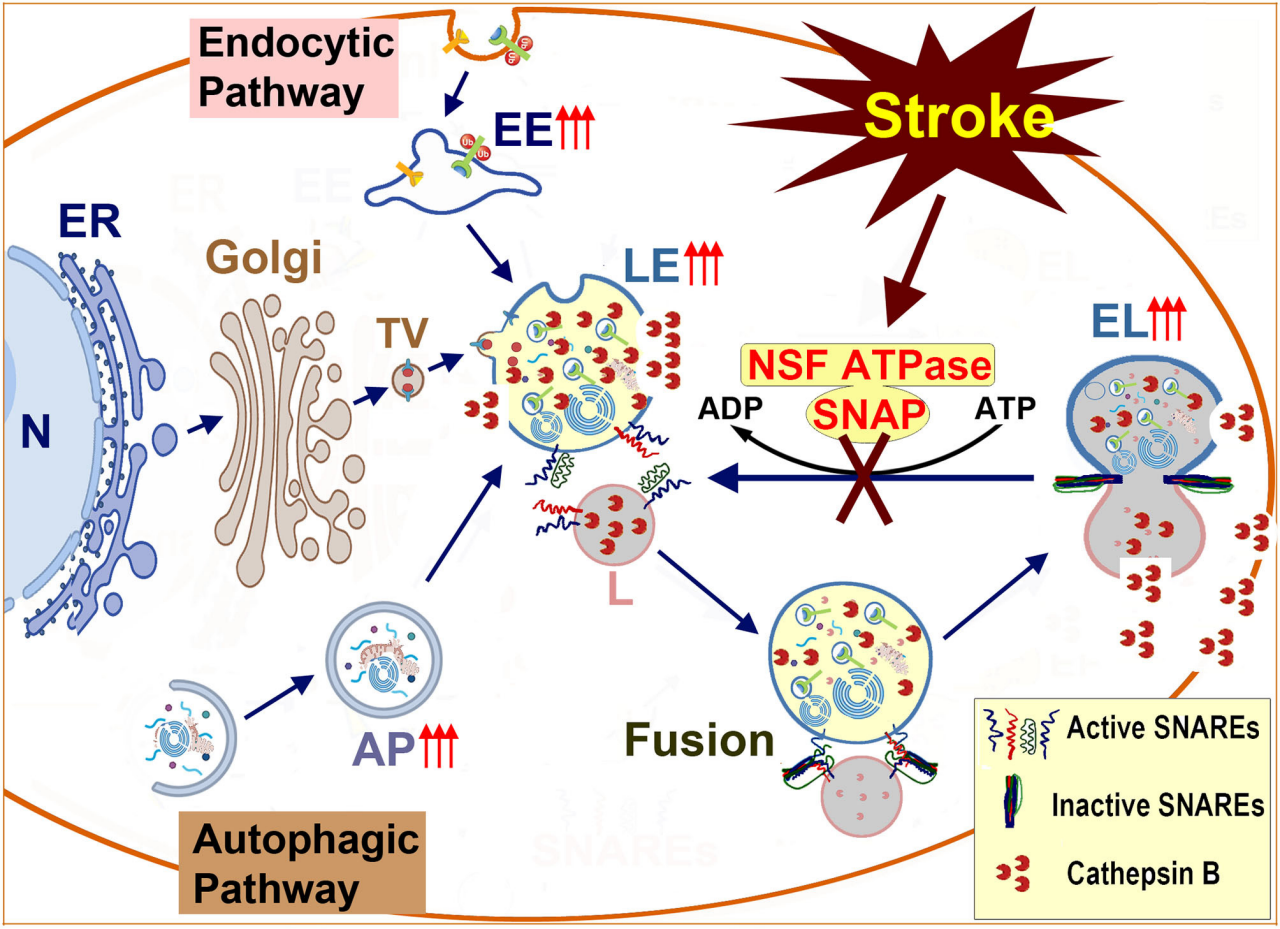
Schematic diagram of endolysosomal trafficking after brain ischemia: endolysosomal hydrolytic enzymes, membrane components, and structural proteins are synthesized on the ER-associated polyribosomes, modified in the ER and the Golgi lumen, and transported to the late endosome. The late endosome also receives incoming cargos from the endocytic and autophagic pathways. The enzyme- and cargo-loaded late endosome fuses with the terminal lysosome to form an endolysosome to digest cargos. After, the endolysosome reform into a terminal lysosome for the next round of fusion. The membrane fusion is mediated by the NSF-SNAP-SNAREs machinery and assisted by other proteins such as HOPS complexes and Rab7. Brain ischemia inactivates NSF ATPase, prevents the regeneration of active SNAREs, and interrupts incoming traffic from both the autophagic and endocytic pathways. This interruption leads to a buildup of damaged LE and EL to release cathepsins such as CTSB and brain ischemia reperfusion injury. N, Nucleus; ER, Endoplasmic Reticulum; Golgi, Golgi apparatus; TV, Transport Vesicle; EE, Early Endosome; AP, Autophagosome; LE, Late Endosome; L, Lysosome; NSF, N-ethylmaleimide Sensitive Factor (ATPase); SNAP, Soluble NSF Attachment Protein; SNARE, SNAP Receptor.

As shown in Figures 1, 7, the LE-to-L fusion is NSF dependent (Mullock et al., 1998). Mutation of the first NSF ATP hydrolysis site (replacement of the aminoacid 329 glutamate with glutamine or E329Q mutation) leads to complete cellular NSF ATPase inactivation via a dominant-negative mechanism (Whiteheart et al., 1994). This dominant-negative inhibition is because the incorporation of a single E329Q NSF into an NSF ATPase hexamer completely eliminates NSF ATPase activity (Whiteheart et al., 1994). Expressing E329Q NSF in cell cultures leads to similar fragmentation of Golgi stacks into dispersed vesicular structures as those observed in post-ischemic neurons (Dalal et al., 2004; Yuan et al., 2018b). Expression of the E329Q NSF dominant-negative mutant in cell cultures also leads to similar NSF deposition as that seen in post-ischemic tissue samples into the two major systems: (i) the Golgi apparatus, and (ii) the endolysosomal system (that are co-immunolabeled with LAMP2) (Dalal et al., 2004). Meanwhile, other subcellular structures such as the ER are minimally affected by expression of E329Q NSF in cell cultures (Dalal et al., 2004). Therefore, the Golgi and endolysosomal systems are primarily damaged by expression of the E329Q NSF dominant-negative mutant (Dalal et al., 2004). These studies suggest that the Golgi and endolysosomal structures are the most dynamic membrane trafficking organelles containing the highest concentrations of NSF, SNAP, and SNARE proteins (Dalal et al., 2004). In comparison, other subcellular organelles such as ER membranes recruit little NSF despite the known ability of SNAREs to bind α-SNAP and NSF in these subcellular organelles (Dalal et al., 2004). These E329Q NSF dominant-negative mutant studies clearly indicate that the Golgi and endolysosomal systems depend on NSF-mediated membrane fusion to maintain their normal structures and functions, whereas other subcellular organelles have only a minimal need for the NSF-SNAP-SNARE machinery to support their normal structures (Dalal et al., 2004). This conclusion is further enforced by NSF knockdown studies. Knockdown of NSF in normal immortalized human kidney (HK2) cells primarily results in: (i) fragmentation of the Golgi apparatus; (ii) impairment of trafficking of lysosomal hydrolases to late endosomes; and (iii) a large scale buildup of EEA1-immunopositive early endosomes and Rab7-immunopositive late endosomes under confocal microscopy, as well as various endolysosomal structures containing whole organelles, membrane whorls, and electron-dense aggregates under EM (Lanning et al., 2018). Knockdown of NSF or α-SNAP also leads to a significant buildup of sealed autophagosomes located in close proximity to lysosomes but not fused with them (Abada et al., 2017; Lanning et al., 2018). All of the NSF inactivation-induced changes (e.g., Golgi fragmentation and buildup of endolysosomal structures) observed in cell cultures can also be found in post-ischemic neurons (Figures 2–5).

Biochemical studies further showed that brain ischemia primarily led to deposition of Golgi and endolysosomal proteins into Triton-insoluble protein aggregates, including Golgi marker proteins (GM130, giantin, and GS28), endosomal marker proteins (syntaxin13 and EEA1), autophagic marker proteins (LC3II and p62), and endolysosomal marker proteins (LAMP1/3 and CTSB) (Liu et al., 2010; Yuan et al., 2018a). In comparison, membrane trafficking proteins unrelated to Golgi and endolysosome systems such as synaptic syntaxin 1 and synaptotagmin1, plasma membrane syntaxin 2, exocytic Rab3, and the ER p97 were unchanged after brain ischemia (Yuan et al., 2018a). Multiple lines of evidence support that NSF inactivation after brain ischemia leads to Golgi apparatus fragmentation and a large-scale buildup of early endosomes, autophagosomes, late endosomes, and endolysosomes (Hu et al., 2000b; Liu and Hu, 2004; Zhang et al., 2006; Yuan et al., 2018b, 2021) (Figure 7).

### Release of Golgi and Endolysosomal Components Into the Extracellular Space After Brain Ischemia

The following observations show support that CTSB, as well as TGN38, p62, and ubiquitin may be released into the extracellular space: (i) CTSB, TGN38, p62, and ubiquitin immunoreactivities that were normally located inside sham-operated control neurons, were found abnormally distributed outsides of neuronal soma in the brain region where selective neuronal death occurs after brain ischemia (Figures 2–5) (Yuan et al., 2021); (ii) cellular contents and endolysosomal structures were found outside of ruptured neurons under EM (Yuan et al., 2021); and (iii) Western blot analysis showed that CTSB protein was significantly increased in the 165,000 g supernatant fraction prepared from post-ischemic brain tissue, but was virtually absent in the same supernatant fraction prepared from sham-operated control brain tissue. This suggests that CTSB might leak out from the endolysosomal compartment into the cytosol or extracellular space (Yuan et al., 2018b, 2021).

### Microscale vs. Large Scale Release of Cathepsins After Brain Ischemia

N-ethylmaleimide sensitive factor depletion leads to an ever-increasing buildup of endolysosomal compartments. A prolonged buildup of endolysosomal compartments leads to endolysosomal membrane damage or rupture, leading to the release of cathepsins (Yuan et al., 2018a). For most endolysosomal dysfunction-related diseases, the mechanisms underlying the endolysosomal damage remains unknown (Wang et al., 2018; de Araujo et al., 2020). Recent results from our laboratory demonstrated that the endolysosomal damage could be caused by the interruption of the fusion between late endosomes and terminal lysosomes and the interruption of the deliveries of lipids, hydrolytic enzymes, and structural proteins from the Golgi to the endolysosomal compartments. These interruptions were caused by NSF inactivation (Yuan et al., 2018a). For these reasons, the endolysosomal compartments were filled with undigested materials and may eventually rupture to release cathepsins after brain ischemia.

There are several studies describing the role of released cathepsins after brain ischemia. Nearly all studies focused on CTSB, while only a couple of studies discussed cathepsins D, E, H, and L. Cathepsin B is the most abundant cathepsin in the brain (Pungercar et al., 2009; Turk et al., 2012). Nitatori et al. (1995) showed that immunoreactivities of cathepsins B, H, and L increased in hippocampal CA1 pyramidal neurons at day three of reperfusion after a brief episode of global brain ischemia in a gerbil model. Nakanishi et al. (1993) showed that levels of cathepsins D and E were unchanged in both the hippocampal and neostriatal neurons before day three of reperfusion after a brief episode of forebrain ischemia. Kohda et al. (1996) showed that the enzymatic activity of CTSB increased while the enzymatic activity of cathepsin L decreased in the CA1 neurons at 24 h after a brief episode of global brain ischemia in a monkey model. Hill et al. (1997) showed that the distribution of CTSB changed from lysosomal to cytoplasmic after a brief episode of global brain ischemia. The studies mentioned above consistently demonstrated that both the levels and activities of CTSB were increased, while those of cathepsins D and E remained unchanged in the hippocampal CA1 neurons destined for death after a brief episode of global brain ischemia in animal models.

Seyfried et al. (1997) showed that increased CTSB immunoreactivity was detected exclusively in the penumbral neurons 2 h post-reperfusion following a 2 h MCAO in rats. Furthermore, continuous intraventricular infusion of stefin A, a weak CTSB inhibitor, before 2 h of MCAO significantly reduced infarct volume, relative to that of a vehicle-treated control group. The authors suggested that increased neuronal CTSB during 2 h of post-reperfusion contributed to neuronal cell death (Seyfried et al., 1997). Anagli et al. (2008) showed that CTSB activity increased predominantly in the ischemic hemisphere after MCAO in rats. Furthermore, post-ischemic treatment with cysteine protease inhibitor 1 (CP-1) reduced infarct volume, neurological deficits, and CTSB activity in the brain. Chaitanya and Babu (2008) showed that the CTSB levels significantly increased in post-ischemic neurons at 1 and 12 h post-reperfusion after MCAO in rats. Ni et al. (2015) showed that focal brain hypoxia caused extensive brain injury in neonatal wild-type mice, but not in CTSB knockout mice. Hossain et al. (2020) showed a time-dependent decrease in CTSD protein levels and activity in the mouse brain after MCAO. The aforementioned studies demonstrate that both the levels and activities of CTSB were upregulated, whereas the level of cathepsin D was decreased in the ischemic region after focal brain ischemia in animal models.

Evidence supports that a microscale amount of CTSB is released from mildly damaged endolysosomal compartments after a brief episode of global brain ischemia. This amount of CTSB release may be limited inside neurons to cause a non-rupture type of cell death after a brief episode of global brain ischemia (Wang et al., 2018; Yuan et al., 2018a). When compared to a brief episode of global brain ischemia (10–15 min), prolonged focal brain ischemia (e.g., 1–2 h) results in a more significant release of CTSB. Our recent work showed that CTSB was released on a large scale from substantially more damaged endolysosomal compartments into the cytoplasm and eventually into the extracellular space (see Figures 3–6). This large-scale CTSB release causes direct tissue destructive infarction (Alu et al., 2020).

In addition to the endolysosomal structures, CTSB is also released from damaged Golgi fragments. Previous cell culture studies show that the 46 kDa pro-CTSB in the Golgi can autocleave its own propeptide to become a 33 kDa active CTSB, which in turn can cleave the propeptide of another pro-CTSB to initiate a chain reaction. This chain reaction eventually leads to a substantially accelerated production and release of active CTSB from damaged Golgi fragments (Pungercar et al., 2009; Turk et al., 2012). Yuan et al. (2018b) also showed that pro-CTSB was significantly reduced while mature CTSB was substantially upregulated in neocortical tissue samples after a brief episode of global brain ischemia. Released CTSB can further damage the Golgi and endolysosomal structures, resulting in a cycle of amplified CTSB and neuronal injury.

Previous studies show that CA-074me and E64d might offer neuroprotection by inhibiting CTSB after brain ischemia (e.g., Yamashima et al., 1998; Xu et al., 2016). However, these treatments increase the endolysosomal CTSB half-life and concentration by about 2–3 fold (Katunuma, 2010). E64d is not a CTSB-specific inhibitor. Numerous previous studies indicate that CA-074 and CA-074me are not CTSB-specific inhibitors either, despite previous claims (Montaser et al., 2002; Mihalik et al., 2004; Wieczerzak et al., 2007; Reich et al., 2009). More importantly, E64d and CA-074me inhibitory effects are dependent on pH. They exhibit strong inhibitory activity at pH 4.5 present in the endolysosomal luminal acidic environment, but are ineffective when CTSB is released into the neutral pH of the cytoplasm or extracellular space (Cathers et al., 2002). The endolysosomal luminal CTSB is required to degrade toxic materials, while released CTSB in the cytosolic and extracellular space is harmful. Therefore, inhibition of endolysosomal luminal cathepsin activities likely leads to deficiency in the endolysosomal degradation activities, causing increased dysfunction of the endocytic and autophagic pathways and more cell death after brain ischemia. An ideal inhibitor would limit its inhibitory capacity to the harmful released CTSB in the cytoplasm and extracellular space, while having minimal impact on the endolysosomal luminal degradation activities.

## Concluding Remarks

It is well-established that a brief episode of cardiac arrest or global brain ischemia leads to delayed neuronal death selectively in most hippocampal CA1 and some neocortical pyramidal neurons (Smith et al., 1984; Hu et al., 2000b; Yuan et al., 2018a). In comparison, stroke or focal brain ischemia with or without reperfusion results in destructive infarction in the brain during the 1 to 24-h post-stroke period (Hu et al., 2001). The mechanism underlying brain ischemia reperfusion injury after both cardiac arrest and stroke remains only partially understood. The latest studies show evidence that brain ischemia inactivates neuronal NSF ATPase. This inactivation results in a cascade of endolysosomal structural damage and the release of digestive enzymes such as CTSB, leading to more structural damage.

There are significant similarities and also differences in the cell death mechanisms in the context of NSF inactivation and endolysosomal structural damage between global and focal brain ischemia animal models. In the global brain ischemia models, milder endolysosomal structural damage or microscale cytosolic CTSB release induces the cell death pathways such as the MOMP cell death pathways (Wang et al., 2018; Yuan et al., 2018b). In comparison, CTSB is released on a large scale from the endolysosomal compartments into the cytoplasm and eventually into the extracellular space after stroke (see Figures 3–6). This large-scale CTSB release results in tissue destruction or infarction (Alu et al., 2020). Due to these differences, treatment strategies against a brief episode of global brain ischemia should target the microscale release of CTSB in the cytosol. In contrast, management of the large-scale CTSB release from both intracellular and extracellular sources is likely to be more effective against stroke brain injury.

Although significant progress has been made in understanding endolysosomal trafficking in post-ischemic neurons, many questions remain. For example, the interruption of endolysosomal trafficking was observed mainly in post-stroke penumbral neurons in which pathology progresses somewhat slowly for observing NSF depletion and accumulation of endolysosomal structures. Therefore, it is unknown if endolysosomal rupture also occurs in rapidly dying neurons of the ischemic core. Additionally, the interruption of endolysosomal trafficking after stroke has primarily been observed in neuronal soma. It remains unclear whether the endolysosomal trafficking defects also occur in the axons and dendrites during the post-ischemic phase.

This review focuses on early intra-neuronal endolysosomal pathological events leading to neuronal death during the first 1 to 24 h after stroke onset. In comparison to early intra-neuronal pathological events, non-neuronal cells change, such as the blood-brain barrier breakdown, microglia activation, monocyte/neutrophil infiltration, and astrogliosis, which mainly occur from 24 h onward after focal brain ischemia (Fifield and Vanderluit, 2020). Changes in endolysosomal trafficking are likely to affect non-neuronal pathological events as well. For example, endolysosomal activities should be significantly upregulated in non-neuronal cells such as reactive microglia and astrocytes or infiltrated macrophages after stroke. The potential differences between a brief episode of global brain ischemia and prolonged focal brain ischemia, as well as between neurons and non-neuronal cells, increases the complexities in developing treatments that target endolysosomal trafficking after brain ischemia.

## Data Availability Statement

The original contributions presented in the study are included in the article/supplementary material, further inquiries can be directed to the corresponding author/s.

## Ethics Statement

All experimental procedures involving animal use were approved by the Animal Use and Care Committees at the University of Miami Millier School of Medicine and University of Maryland School of Medicine.

## Author Contributions

KH wrote parts 1 and 2 of the manuscript. BH planned the conception of the review and wrote part 3 of the manuscript. BG, LS, AA, HT, and CL contributed to the proofreads and revisions of the manuscript. All authors contributed to the article and approved the submitted version.

## Funding

This work was supported by National Institutes of Health (NIH) grants: NS36810, NS40407, NS097875, and NS102815 to BH; by Veteran Affair Merit grant: I01BX001696; and by American Heart Association 0940042N-5 to BH.

## Conflict of Interest

The authors declare that the research was conducted in the absence of any commercial or financial relationships that could be construed as a potential conflict of interest.

## Publisher's Note

All claims expressed in this article are solely those of the authors and do not necessarily represent those of their affiliated organizations, or those of the publisher, the editors and the reviewers. Any product that may be evaluated in this article, or claim that may be made by its manufacturer, is not guaranteed or endorsed by the publisher.
